# The Aberrant DNA Methylation Profile of Human Induced Pluripotent Stem Cells Is Connected to the Reprogramming Process and Is Normalized During *In Vitro* Culture

**DOI:** 10.1371/journal.pone.0157974

**Published:** 2016-06-23

**Authors:** Lenka Tesarova, Pavel Simara, Stanislav Stejskal, Irena Koutna

**Affiliations:** 1 Centre for Biomedical Image Analysis, Faculty of Informatics, Masaryk University, Brno, Czech Republic; 2 International Clinical Research Center, St. Anne’s University Hospital Brno, Brno, Czech Republic; University of Newcastle upon Tyne, UNITED KINGDOM

## Abstract

The potential clinical applications of human induced pluripotent stem cells (hiPSCs) are limited by genetic and epigenetic variations among hiPSC lines and the question of their equivalency with human embryonic stem cells (hESCs). We used MethylScreen technology to determine the DNA methylation profile of pluripotency and differentiation markers in hiPSC lines from different source cell types compared to hESCs and hiPSC source cells. After derivation, hiPSC lines compromised a heterogeneous population characterized by variable levels of aberrant DNA methylation. These aberrations were induced during somatic cell reprogramming and their levels were associated with the type of hiPSC source cells. hiPSC population heterogeneity was reduced during prolonged culture and hiPSCs acquired an hESC-like methylation profile. In contrast, the expression of differentiation marker genes in hiPSC lines remained distinguishable from that in hESCs. Taken together, *in vitro* culture facilitates hiPSC acquisition of hESC epigenetic characteristics. However, differences remain between both pluripotent stem cell types, which must be considered before their use in downstream applications.

## Introduction

Human pluripotent stem cells (hPSCs) have potential applications in regenerative medicine. Initial research with human embryonic stem cells (hESCs) [1] has been revolutionized by the ability to reprogram somatic cells into human induced pluripotent stem cells (hiPSCs) [2–4]. However, the utility of hPSCs is limited by variations in the genome stability of hPSC lines and in their differentiation potential [5–9]. These variations result from genetic and epigenetic differences among hPSC lines coming from genetic background and prolonged culture in hESCs and, in addition, from the reprogramming process in hiPSCs. During somatic cell reprogramming, genes that control pluripotency are introduced to induce pluripotent cellular properties [2–4]. The process is accompanied by epigenomic transformation, generating hiPSCs with molecular and functional characteristics similar to hESCs. Previous research comparing hiPSCs with hESCs has revealed both similarities and differences with regard to the transcriptome, genome stability, histone modification and DNA methylation [10–16].

DNA methylation is an epigenetic mechanism that regulates important biological processes, including stabilization of the pluripotent state in hPSCs, hPSC differentiation and cellular reprogramming [17, 18]. The DNA methylomes of hESCs and hiPSCs have been shown to be similar on a global scale [13], though several studies have identified differences in the DNA methylation status between hESC and hiPSC lines and among hiPSC lines [13, 14, 19, 20]. hiPSCs exhibit a unique methylation profile that is attributable to both somatic memory and aberrant DNA methylation [13, 19, 21–23]. Somatic memory results from the failure to completely reprogram source cell methylation patterns, which occurs late during the reprogramming process and may remain incomplete in hiPSC lines. This incompleteness includes the insufficient silencing of source cell lineage-specific genes, as demonstrated by DNA hypomethylation of relevant loci [12–14, 22–24], and the simultaneous inactivation of genes specific for other tissues, which is associated with hypermethylation [12, 13, 18, 21, 22]. hiPSC source cell memory contributes to the methylome differences between hiPSC and hESCs and among hiPSCs from different source cells and may skew the differentiation potential of hiPSCs, favouring the source cell lineage [12, 22, 24]. The other source of the hiPSC methylation profile is aberrant DNA methylation induced during reprogramming; such methylation is not observed in the original source cells or equivalent hESCs. Hypermethylation is the predominant form of this methylation abnormality [14], and DNA methyltransferase DNMT3B, which is upregulated in hiPSCs [25], may contribute to acquired hypermethylation [26]. Other research has suggested that hypomethylation prevails over hypermethylation in reprogramming-induced methylation aberrancies in CpG dinucleotides [13]. Differentially methylated regions between hESCs and hiPSC fall into two categories. These differences can be specific for individual hiPSC lines and demonstrate methylation variability among lines [13], and they can be common to multiple hiPSC lines, suggesting their predisposition toward aberrant reprogramming. Hot spots of genes with failed epigenomic reprogramming, which are common in hiPSCs, have been identified in every hiPSC line examined [13, 14, 20], and a unique DNA methylation signature that distinguishes between hiPSCs and hESCs has been identified [26]. Variations in the hiPSC DNA methylome can generally be reduced through culturing [20]. In contrast, other research suggests that differentially methylated regions in hiPSCs cannot be erased by passaging and can be transmitted to differentiating cells, potentially altering their properties [13, 14, 27]. These findings indicate that the reprogramming variability of hiPSCs and the possible persistency of disrupted DNA methylation must be considered before hiPSCs can be successfully used for downstream applications.

We used MethylScreen technology, developed by Holemon *et al*. [28], to analyze the DNA methylation profile at transcription start site (TSS)-associated CpG islands for selected pluripotency and differentiation markers. We compared the profiles of hiPSC lines derived from three different source cells by different reprogramming methods with the profiles of hESC lines and hiPSC source cells. The aim was to determine the extent of aberrant DNA methylation and its variation in derived hiPSC lines intended for *in vitro* differentiation studies.

## Materials and Methods

### Human Cell Cultures

CBIA hiPSC lines (Table 1) were generated in our laboratory. They were derived from neonatal dermal fibroblasts (NDFs), adult dermal fibroblasts (ADFs) and CD34^+^ cells from peripheral blood mononuclear cells (PBMCs) using i) lentivirus containing Oct-4, Klf4, SOX-2, and c-Myc factors (Human STEMCCA Constitutive Polycistronic Lentivirus Reprogramming Kit; Millipore, Darmstadt, Germany; cell line CBIA-1); ii) Sendai virus containing Oct-4, Klf4, SOX-2, and c-Myc factors (CytoTuneTM-iPS Reprogramming Kit; Thermo Fisher Scientific, Waltham, MA; cell lines CBIA-3, CBIA-5); and iii) episomal vectors containing Oct-4, Klf4, SOX-2, and c-Myc and Lin28 factors (Epi5^TM^ Episomal iPSC Reprogramming Kit; Thermo Fisher Scientific; cell lines CBIA-7, CBIA-11) according to the manufacturer’s instructions. Source cell manipulation and the reprogramming procedure was described previously [29], ADFs were treated in the same manner as NDFs. Immunofluorescence analysis of CBIA hiPSC lines with pluripotency markers *OCT4*, *SOX2*, *NANOG*, and *SSEA4* was performed as described [29] and all the lines were positive for these markers (S1A Fig). Teratoma forming assay proved differentiation potential of the hiPSCs into all three germ layers (S1B Fig). Other hiPSC lines (IPSCE, IPSCF and AM13 derived from NDFs by STEMCCA lentivirus reprogramming) and hESC lines (CCTL-12 and CCTL-14) [30] were kindly provided by the Department of Biology, Faculty of Medicine, Masaryk University, Brno, Czech Republic.

**Table 1:** hPSC lines used in this study.

hPSC group	Cell line designation
hESC lines	CCTL-12; CCTL-14
hiPSC lines from NDFs	AM13; CBIA-1; IPSCE; IPSCF
hiPSC lines from ADFs	CBIA-5; CBIA-7
hiPSC lines from PBMC CD34^+^ cells	CBIA-3; CBIA-11

hPSCs were maintained on irradiated mouse embryonic fibroblasts in DMEM/F12 (1:1) medium supplemented with 20% knockout serum replacement, 2 mM L-glutamine, 100 μM non-essential amino acids, 100 U/mL penicillin-streptomycin, 0.1 mM 2-mercaptoethanol and 10 ng/mL recombinant human basic fibroblast growth factor (all from Thermo Fisher Scientific). hPSC colonies were manually passaged every 7 days onto fresh feeder cells. For the purpose of this study, passage numbers up to twenty were considered “low”, passage numbers greater than thirty-five were considered “high”. The cells were feeder depleted by culturing on Geltrex^TM^ (Thermo Fisher Scientific) in Essential 8^TM^ medium (Thermo Fisher Scientific) prior to both methylation analysis and gene expression studies. NDFs (Human Dermal Fibroblasts, neonatal; Thermo Fisher Scientific) and ADFs (derived from the ear skin of an 8-year-old boy; kindly provided by the National Tissue Centre, Brno, Czech republic) were maintained in DMEM supplemented with 20% foetal bovine serum, 2 mM L-glutamine, 100 μM non-essential amino acids, 100 U/mL penicillin-streptomycin and 0.1 mM 2-mercaptoethanol (all from Thermo Fisher Scientific). CD34^+^ cells were isolated from PBMCs and expanded as described previously [29]. Acute myeloid leukaemia cell line KG-1 (DSMZ, Braunschweig, Germany) was maintained in RPMI 1640 medium supplemented with 10% foetal bovine serum.

Spontaneous differentiation of hiPSCs was induced by the formation of spin embryoid bodies (EBs) in BPEL medium, as previously described [31, 32]. Briefly, hiPSCs were dissociated to a single-cell suspension via TrypLE Select (Thermo Fisher Scientific) treatment and plated at 3000 cells per well into low-attachment, round-bottom, 96-well plates. After two days, the generated EBs were pooled and cultured in suspension in a low-attachment 35-mm culture dish for nineteen days in BPEL medium; the medium was exchanged every third day.

### DNA Isolation and Restriction

Genomic DNA was isolated using DNeasy Blood & Tissue Kit (Qiagen, Hilden, Germany) according to the manufacturer’s protocol. Restriction enzymes HhaI and McrBC were used to digest 1 μg of genomic DNA in four restriction reactions. Each 30 μL reaction contained 250 ng of genomic DNA, 1x NEBuffer 2, 100 μg/mL bovine serum albumin, 1 mM guanosine-5'-triphosphate and HhaI alone (20 U, Rs reaction), McrBC alone (10 U, Rd reaction), HhaI and McrBC combined (Rsd reaction) or water instead of enzymes (R0 reaction). Digestions were incubated at 37°C for 6 hours followed by enzyme inactivation at 65°C for 20 minutes. The enzymes, NEBuffer 2, bovine serum albumin, and guanosine-5'-triphosphate were purchased from New England Biolabs (Ipswich, MA, USA).

### MethylScreen Reactions

Restricted samples were analysed by quantitative real-time PCR with locus-specific PCR primers and SYBR Green fluorescent dye for the detection of amplified products. A set of PCR primers was designed to amplify genomic DNA at the TSS-associated CpG islands of eleven genes (Table 2). The PCR amplicons were 390 bp in length (average) and contained 5 HhaI restriction sites and 39 CpG dinucleotides. The PCR amplification was performed in a 50 μL volume with 1x FastStart Universal SYBR Green Master (Roche Diagnostics, Mannheim, Germany), 300 nM of each primer and 1.25 μL digested template DNA using the 7500 Real-Time PCR System (Applied Biosystems, Foster City, CA, USA). The PCR conditions were as follows: 50°C for 2 min, 95°C for 2 min, and 45 cycles of 95°C for 15 s and 65°C for 1 min. Each qPCR reaction was performed in duplicate in a 96-well plate. To verify the amplification of desired products, melting curve analysis was performed after the reaction. Standard curve data were obtained from 5-point serial dilutions of genomic DNA, and the amplification efficiency (AE) was calculated.

**Table 2:** PCR primers used for MethylScreen reactions.

Primer	Sequence (5´-3´)	Product size (bp)	Numberof CpGs	Number of HhaI restriction sites
CD34-F	GTGAGACTCTGCTCTGCTGTTC	361	33	7
CD34-R	GGGGAGCTCAAGTTAGTAGCAG			
FOXA2-F	CTAGGTGAGAGGTAGCCGCAG	367	39	7
FOXA2-R	AGAGAATGAGCACTGAGAGCG			
GATA2-F	ACTAAGCGGCACAATCAGGAC	400	31	4
GATA2-R	TTGGGCTTCTTAGGCGTGC			
PAX6-F	AGCCACGGTTCCCTTTTCAA	413	36	7
PAX6-R	CAGCACAGAAACTTGCACCC			
RUNX1-F	CAAGCTAGGAAGACCGACCC	424	45	5
RUNX1-R	AATCGGCTTGTTGTGATGCG			
SOX2-F	CCTGATTCCAGTTTGCCTCTCT	391	45	4
SOX2-R	CATCTTGGGGTTCTCCTGGG			
SOX17-F	CCAGCTCCGGCTAGTTTTCC	446	46	4
SOX17-R	CTGGTCGTCACTGGCGTATC			
T-F	TCTGGAAAAGGAAGGTCCGC	402	29	5
T-R	CTGGGCTCCCGTTTTAGGAG			
TCERG1L-F	CACCCCTCTGTGAATTCGTC	372	33	5
TCERG1L-R	GTTTCCTGGGTTTAGCGACC			
TSPYL5-F	CCCCGAGACTCTGGTACTGT	359	52	8
TSPYL5-R	GAGTCCGCGCGAGATGG			
UTF1-F	AATTCCGACACCCATTCCCG	350	36	2
UTF1-R	GCAAAGACTGGCTTCGGTTG			

### Calculations of DNA Methylation Occupancy

Ct values from R0, Rs, Rd and Rsd restrictions were used to calculate the initial amount of DNA prior to PCR (AE^-ct^). The DNA methylation occupancy (%) was calculated as follows: hypermethylated (HM) fraction = Rs/(R0-Rsd) x 100; unmethylated (UM) fraction = Rd/(R0-Rsd) x 100; intermediately methylated (IM) fraction = 1-HM-UM. If ∆Ct(Rs-R0) < 1.0 or ΔCt(Rd-R0) < 1.0, the following formulas were used to calculate the fraction of hypermethylated or unmethylated DNA, respectively: HM = 1—UM, UM = 1—HM. The fraction of DNA copies resistant to enzyme digestion was calculated as Rsd/R0 [33].

### Quantitative Real-Time PCR

Total RNA was prepared with RNeasy Mini Kit (Qiagen) according to the manufacturer’s protocol and treated with RNase-Free DNase (Qiagen). An RNA aliquot was reverse-transcribed into cDNA using SuperScript II Reverse Transcriptase (Thermo Fisher Scientific) and Oligo(dT)_23_ primers (Sigma-Aldrich, St. Louis, MO) with the addition of RNasin RNase Inhibitor (Promega, Mannheim, Germany). The cDNA template was amplified using specific primers for pluripotency and differentiation markers (Table 3). GAPDH expression was used as the control. All experiments were performed in triplicate using FastStart Universal SYBR Green Master (Roche Diagnostics) and a 7500 Real-Time PCR System (Applied Biosystems). The PCR conditions were: 50°C for 2 min, 95°C for 2 min, and 40 cycles of 95°C for 15 s and 60°C for 1 min. Relative gene expression was quantified using the AE^−∆∆C*t*^ method.

**Table 3:** PCR primers used for gene expression analysis.

Primer	Sequence (5´-3´)	Product size (bp)
CD34-F	GCGCTTTGCTTGCTGAGT	67
CD34-R	GGGTAGCAGTACCGTTGTTGT	
FOXA2-F	GAGCGGTGAAGATGGAAGG	118
FOXA2-R	GTGTTCATGCCGTTCATCC	
GAPDH-F	CTGACTTCAACAGCGACAC	105
GAPDH-R	CAAATTCGTTGTCATACCAGGA	
GATA2-F	GCACAATGTTAACAGGCCAC	127
GATA2-R	CATGCACTTTGACAGCTCCT	
PAX6-F	TCACCATGGCAAATAACCTG	71
PAX6-R	CAGCATGCAGGAGTATGAGG	
POU5F1-F	GGTTCTATTTGGGAAGGTATTCAG	160
POU5F1-R	GGTTTCTGCTTTGCATATCTCC	
NANOG-F	AGATGCCTCACACGGAGACT	127
NANOG-R	TTTGCGACACTCTTCTCTGC	
RUNX1-F	ATCGCTTTCAAGGTGGTGG	163
RUNX1-R	CACTTCGACCGACAAACCT	
SOX2-F	TGCAGTACAACTCCATGACCA	123
SOX2-R	ACTTGACCACCGAACCCA	
SOX17-F	CGCCGAGTTGAGCAAGAT	113
SOX17-R	GGTGGTCCTGCATGTGCT	
T-F	AACTCCTTGCATAAGTATGAGCC	141
T-R	TTTAAGAGCTGTGATCTCCTCGT	
UTF1-F	CGACATCGCGAACATCCT	122
UTF1-R	TGCCCAGAATGAAGCCC	

## Results

### Detection limits of MethylScreen Technology

**Fig 1:**
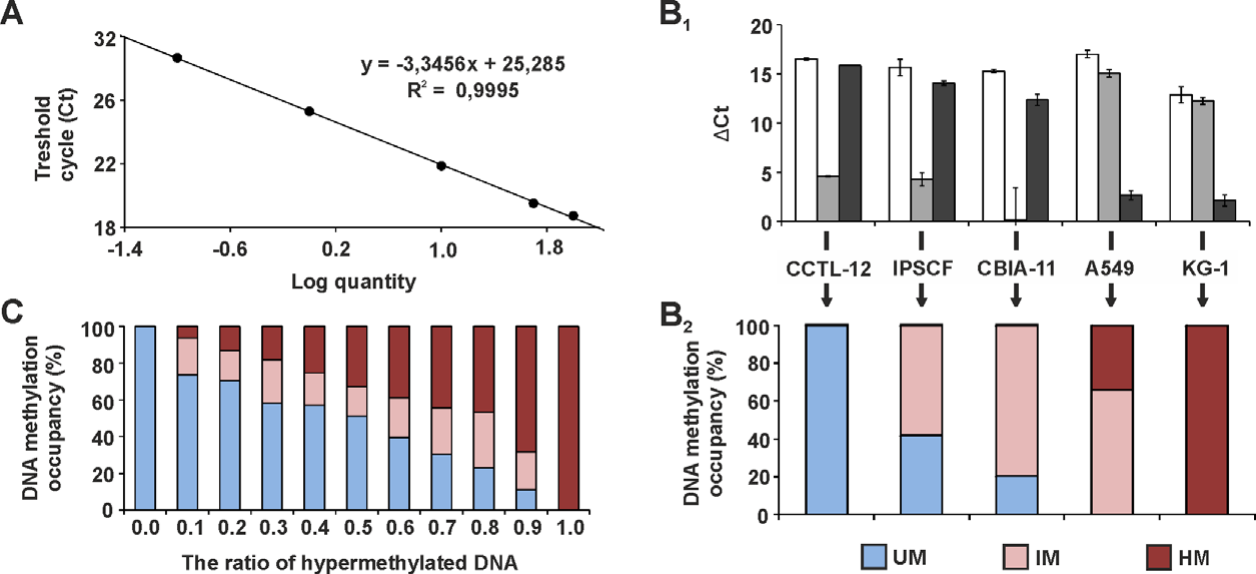
MethylScreen technology principles and data interpretation. (A) Representative qPCR standard curve for PAX6 obtained from 100, 50, 10, 1 and 0.1 ng of control DNA per reaction. (B_1_) *UTF1* MethylScreen qPCR results for CCTL-12, IPSCF, CBIA-11, A549 and KG-1 genomes. Changes in c_t_ between enzyme treated and non-treated templates are depicted: Rs-R0 is represented by white columns (HhaI reaction), Rd-R0 is represented by grey columns (McrBC reaction), and Rsd-R0 is represented by black columns (both HhaI and McrBC reaction). (B_2_) *UTF1* DNA methylation profile for five cell lines. c_t_ values from four restriction reactions (B_1_) were converted to DNA methylation occupancy, expressed as the percentage of unmethylated (UM), intermediately methylated (IM) and hypermethylated (HM) DNA. (C) MethylScreen results obtained from sample mixtures with an increasing ratio of hypermethylated DNA (0–100%, x-axis). The colour legend is identical for (B_2_), and the values are averages for *PAX6* and *TSPYL5* genes after serial mixing of CCTL-14 (unmethylated) and A549 (hypermethylated) samples and NDFs (unmethylated) and CCTL-14 (hypermethylated) samples, respectively.

The detection limits of the MethylScreen technology during the analysis of DNA methylation profiles were determined. The technology is based on the combined digestion of two enzymes with opposite sensitivity to DNA methylation at CpG-rich regions [28]. Sample genomic DNA was treated in four restriction reactions during the analysis. The first reaction (Rs) consisted of the methylation-sensitive restriction enzyme HhaI cutting the DNA at GCGC sequences without methylcytosine. The second reaction (Rd) consisted of the methylation-dependent restriction enzyme McrBC recognizing a pair of methylcytosines in the form of 5’…A/G^m^C(N_40-3000_)A/G^m^C…3’. The third reaction (R0), without any enzyme, determined the total amount of input DNA in the analysis. Both HhaI and McrBC were present in the fourth reaction (Rsd) to assess the amount of DNA amenable to analysis. The amount of DNA remaining after each restriction was quantified using qPCR with primers amplifying the region of interest. For this study, eleven genes were selected to represent markers for pluripotent cells (*UTF1*, *SOX2*), embryonic germ layers (*T*, *FOXA2*, *PAX6*) and blood progenitors (*SOX17*, *RUNX1*, *GATA2*, *CD34*) as well as genes with described DNA methylation variability in hPSCs (*TCERG1L*, *TSPYL5*) [13, 14, 20]. PCR primers were designed to amplify genomic DNA at the TSS-associated CpG islands of these genes. The PCR amplicons were 350–446 bp in length and contained as many as possible sites for both HhaI and McrBC to maximize the sensitivity of methylation detection (Table 2). The PCR product specificity was verified by melting curve analysis and gel electrophoresis after the reactions. The AE was determined from standard curve data (Fig 1A) and applied to calculations of the DNA methylation profile. The profile was calculated from the ratio of Ct values in R0, Rs, Rd and Rsd reactions; it is presented as a percentage of the unmethylated, intermediately methylated and hypermethylated fractions of the total DNA (Fig 1B). The assay sensitivity was conditioned by the size of the DNA population amenable to restriction and was represented by ∆Ct between the Rsd and R0 reactions. This analytical window was from 13.3 to 21.1 (average for all analysed cell lines) for individual genes. The sensitivity of the detection of DNA methylation was verified by analysing samples with an increasing ratio of DNA from cell lines with fully hypermethylated and unmethylated DNA for a given gene (Fig 1C). The ratio of unmethylated and hypermethylated DNA fraction was preserved with the addition of up to a 30% intermediately methylated DNA fraction.

### Derived hiPSC Lines Are Characterized by Aberrant DNA Methylation Profiles

**Fig 2:**
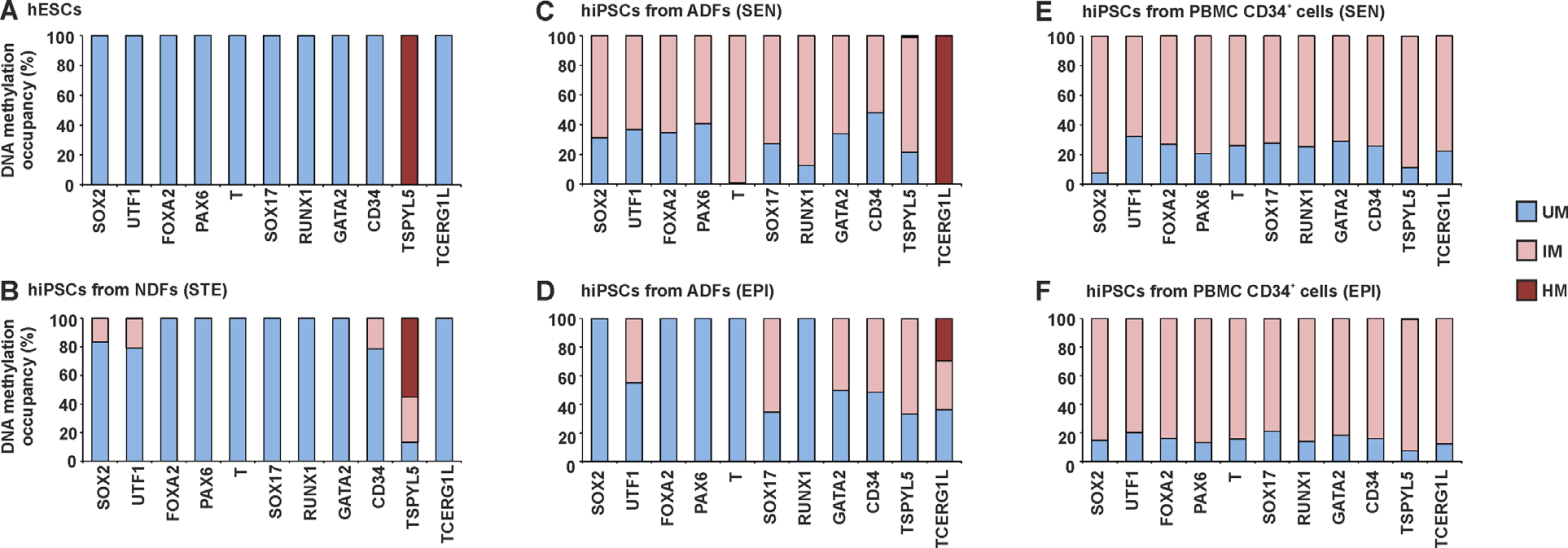
DNA methylation profile of selected genes in hPSC lines. DNA methylation occupancy is reported as the percentage of unmethylated (UM), intermediately methylated (IM) and hypermethylated (HM) DNA. The profile is shown for hESC lines (A), hiPSC lines from NDFs (B), hiPSC lines from ADFs (C, D) and hiPSC lines from PBMC CD34^+^ cells (E, F) generated by STEMCCA lentivirus (STE), Sendai virus (SEN) and the episomal vector (EPI) reprogramming. Values are the averages from hPSC lines within the line group where appropriate.

MethylScreen technology was used to determine the DNA methylation profile in hPSC lines. hESCs were used to obtain a control DNA methylation profile of established hPSCs. hiPSCs were analysed early after derivation, with a passage number of less than twenty. hiPSC lines were derived from three different cell sources, NDFs, ADFs, and PBMC CD34^+^ cells, using three different reprogramming techniques (STEMCCA lentivirus, Sendai virus, and an episomal vector). In the hESC lines, all analysed genes were unmethylated, except for the full hypermethylation of *TSPYL5* (Fig 2A). hiPSCs generated from NDFs by STEMCCA lentivirus reprogramming were characterized by an hESC-like methylation profile, with the exception of decreased *TSPYL5* hypermethylation and up to 30% intermediately methylated DNA for *SOX2*, *UTF1* and *CD34* (Fig 2B). A variable level of intermediately methylated DNA of analysed genes was detected in hiPSC lines derived from ADFs (Fig 2C and 2D) and PBMC CD34^+^ cells (Fig 2E and 2F) after Sendai virus (Fig 2C and 2E) and the episomal vector (Fig 2D and 2F) reprogramming. In addition, hiPSCs from ADFs displayed 100% (Sendai virus reprogramming) and 30% (episomal vector reprogramming) hypermethylation of *TCERG1L*. These results suggest that the level of aberrant DNA methylations in hiPSCs is associated with the source cells used for their derivation.

**Fig 3:**
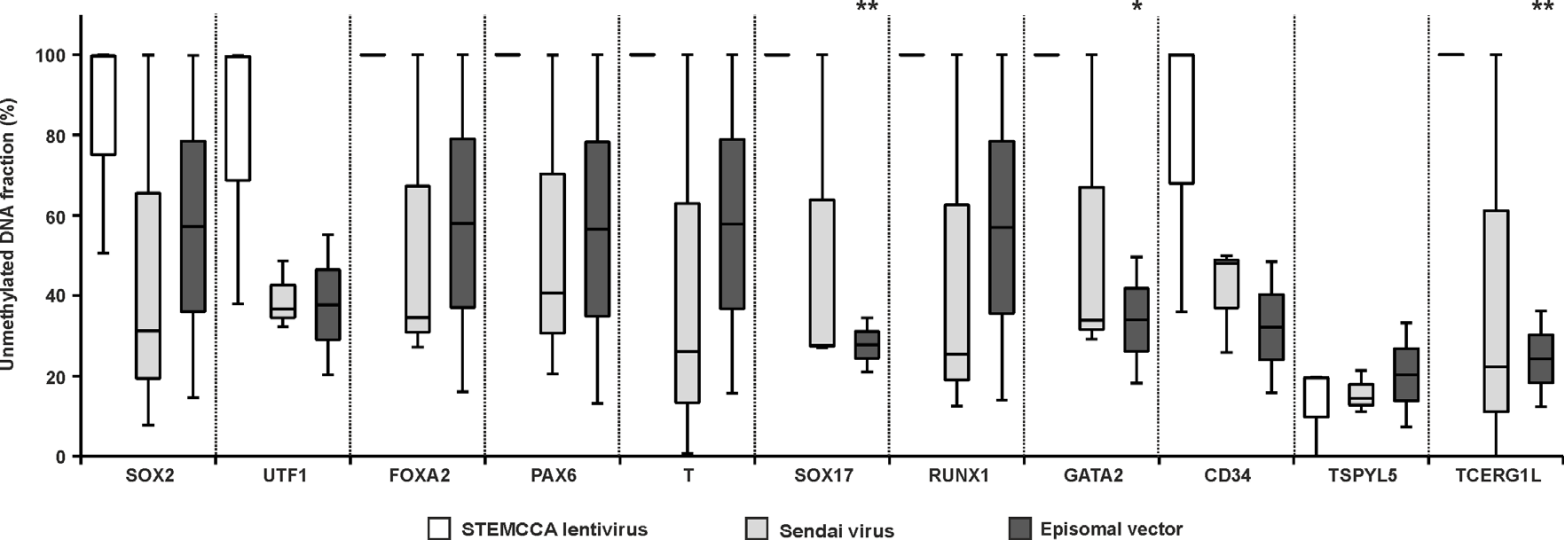
DNA methylation level in hiPSC lines derived via different reprogramming techniques. The fraction of unmethylated DNA, reported as the percentage of the analysed DNA population, was determined for hiPSC lines with low passage numbers derived by STEMCCA lentivirus (white), Sendai virus (light grey) and Episomal vector (dark grey) reprogramming. Boxes extend from the 25^th^ to the 75^th^ percentiles; the line in the middle and the bars represent the population median and both the minimal and maximal values, respectively. Asterisks above the grey and black boxes indicate a significant difference between white and grey and white and black boxes, respectively (* for p<0.05, ** for p<0.005).

DNA methylation profile was compared among hPSC lines generated by different reprogramming techniques regardless their source cell type. After STEMCCA lentivirus reprogramming, the level of unmethylated DNA was high for the majority of analysed genes (Fig 3). However, this parameter was decreased in hiPSCs generated using Sendai virus as well as the episomal vector reprogramming, with an increased fraction of intermediately methylated DNA. Nonetheless, for the majority of genes the differences were characterized by a low degree of significance due to sample variability; therefore the association between the level of DNA methylation in hiPSCs and the reprogramming method was not confirmed.

**Fig 4:**
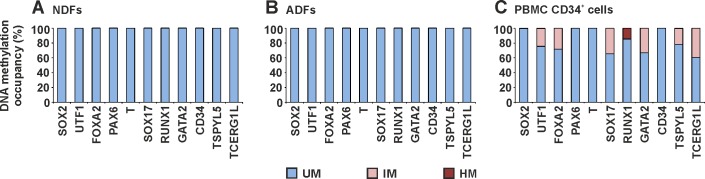
DNA methylation profile of selected genes in hiPSCs source cells. DNA methylation occupancy is reported as the percentage of unmethylated (UM), intermediately methylated (IM) and hypermethylated (HM) DNA. The profile is shown for NDFs (A), ADFs (B), and PBMC CD34^+^ cells (C).

The DNA methylation profiles of the source cells used for hiPSC derivation were analysed to determine the origin of aberrant hiPSC DNA methylation. Unmethylated DNA of all analysed genes was found in both NDFs and ADFs (Fig 4A), and PBMC CD34^+^ cells were also characterized by a dominant fraction of unmethylated DNA (Fig 4B). These results demonstrate that the DNA methylation profile of source cells changed during the reprogramming process and that aberrant DNA methylation was induced.

### Reprogramming-induced Aberrant DNA Methylation in hiPSCs Is Reduced During Culturing

**Fig 5:**
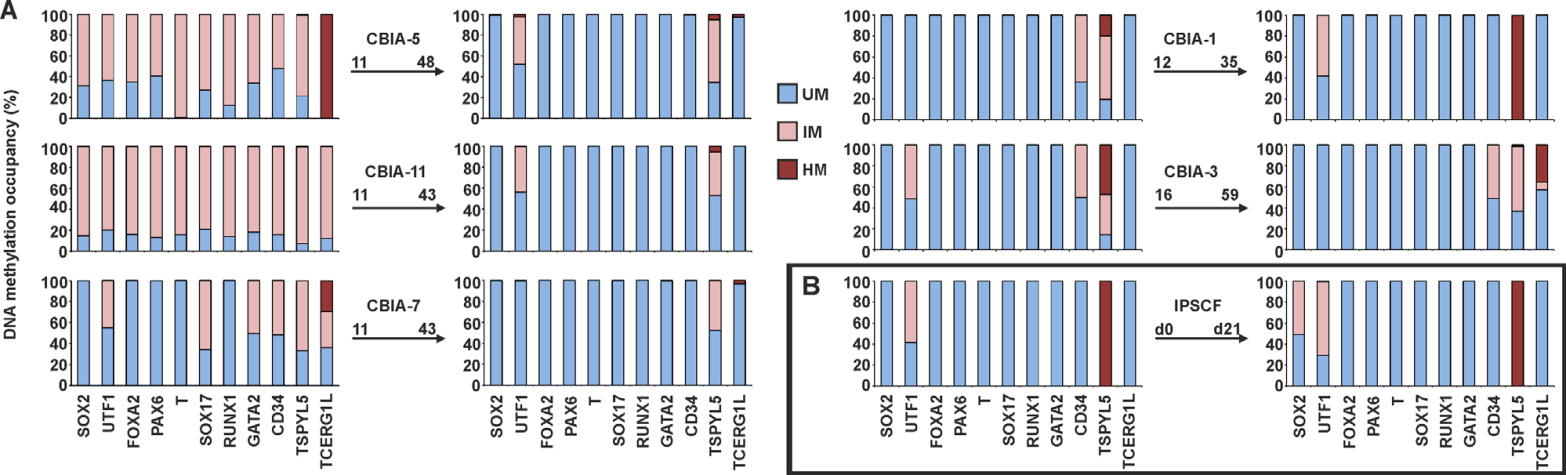
Changes in the DNA methylation profile during prolonged hiPSC culture and differentiation. DNA methylation occupancy is reported as the percentage of unmethylated (UM), intermediately methylated (IM) and hypermethylated (HM) DNA. (A) An arrow connects corresponding DNA methylation profiles of the cell line in low and high passages. Passage numbers and the hiPSC line name are reported above the arrow. (B) DNA methylation profile of the IPSCF line before (d0) and after three weeks of spontaneous differentiation (d21).

The effect of prolonged *in vitro* culturing on the DNA methylation profile was investigated through the analysis of hiPSC lines after several weeks of continuous passage. The average difference between low and high passages was 33.2 (±6.9) passages (Fig 5A). After this period, all analysed hiPSC lines were characterized by an hESC-like DNA methylation profile. With a significant fraction of intermediately methylated DNA after derivation, hiPSC lines decreased their methylation load during culturing. After this normalization of the DNA methylation profile, differences between hiPSC lines with high passage numbers and hESC lines were limited to a decreased fraction of *TSPYL5* hypermethylation and up to a 30% intermediate methylation as well as the hypermethylation of *UTF1*, *CD34* and *TCERG1L* in some hiPSC lines. Spontaneous hiPSC differentiation, in the form of spin EBs, was induced in an hiPSC line with an hESC-like DNA methylation profile. After three weeks of differentiation, the profile did not change, though 50% intermediately methylated DNA of *SOX2* was acquired (Fig 5B).

### Gene Expression Differences in hiPSCs Compared with Source Cells and hESCs

**Fig 6:**
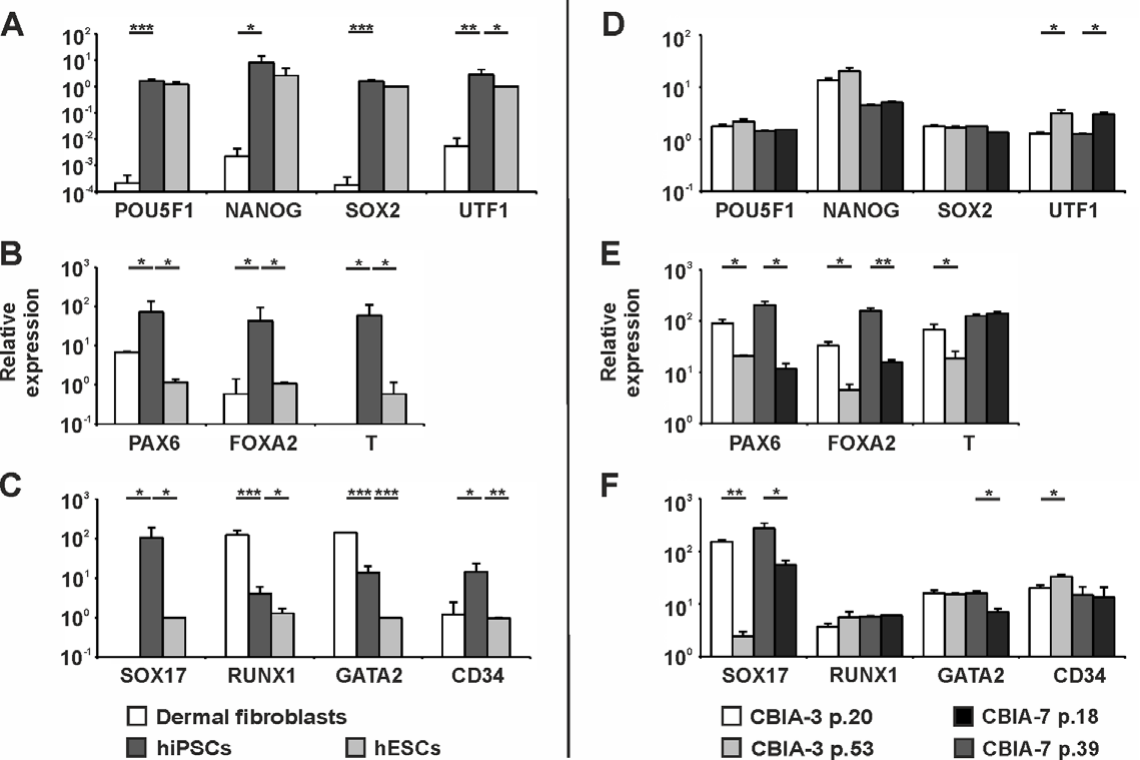
Gene expression levels in hPSCs and dermal fibroblasts, as determined by quantitative real-time PCR. Expression levels of eleven genes were compared i) among the hiPSC lines, hESC lines and dermal fibroblasts (NDFs and ADFs together) (A-C) and ii) within hiPSC lines between low and high passage numbers (D-F). Analysed hiPSC lines and the passage numbers are reported in the graph legend. The values are relative to the values from hESCs (set at 1). Values are the mean + SD, and asterisks indicate the significance between indicated samples: *p < 0.05; **p < 0.005; ***p < 0.0005.

qRT-PCR was used to compare the gene expression of pluripotency and differentiation markers among the hESC lines, hiPSC lines and dermal fibroblasts (ADFs and NDFs) (Fig 6). The majority of genes were activated during hiPSC derivation, and their expression was significantly greater in hiPSCs compared with dermal fibroblasts (Fig 6A–6C). In contrast, *RUNX1* and *GATA2* transcripts were more abundant in dermal fibroblasts (Fig 6C). Pluripotency markers *POU5F1*, *NANOG* and *SOX2* were actively transcribed in both hESCs and hiPSCs (Fig 6A). Differences between these two hPSC groups were found for all other analysed markers, with significantly greater expression levels in hiPSCs (Fig 6A–6C). The expression dynamic of pluripotency and differentiation markers during prolonged culturing was evaluated in CBIA-3 and CBIA-7 hiPSC lines with low and high passage numbers (Fig 6D–6F). Pluripotency markers (*POU5F1*, *NANOG*, *SOX2*) and *RUNX1* were characterized by constant expression levels. Differentiation markers *PAX6*, *FOXA2*, *T*, *GATA2* and *SOX17* demonstrated decreased expression in cells with high passage numbers, whereas the expression of *UTF1* and *CD34* (for CBIA-3) increased during culturing.

**Fig 7:**
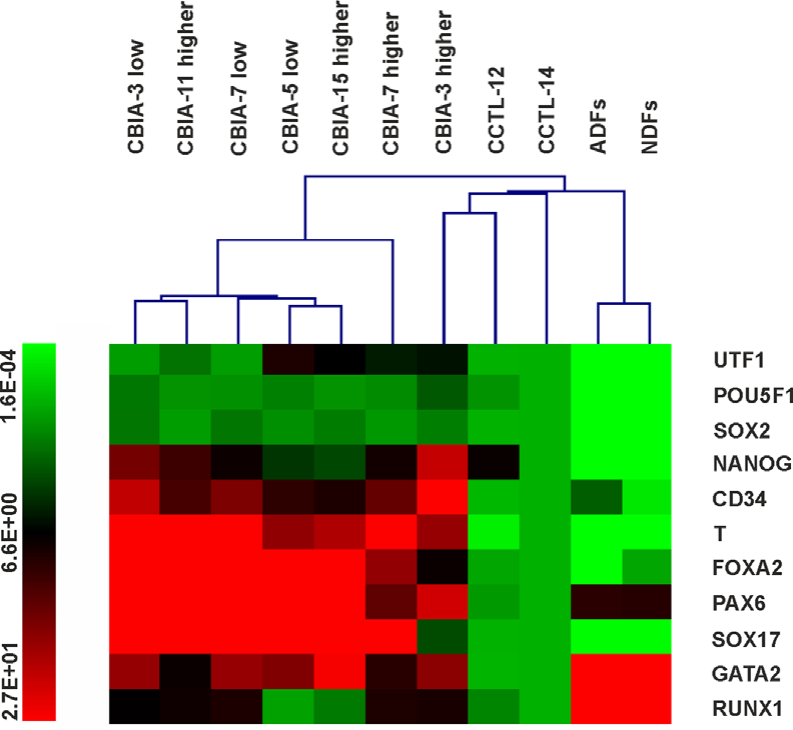
Hierarchical clustering of hPSC lines and dermal fibroblasts based on quantitative real-time PCR data. The colour-coded scale on the left of the picture indicates the expression value for each gene; low expression is represented by green, and high expression is represented by red. The values are relative to the values from CCTL-14 (set at 1).

Hierarchical clustering analysis demonstrated that hiPSCs, hESCs and dermal fibroblasts can clearly be discriminated based on their gene expression profiles (Fig 7). The only exception was the fact that line CBIA-3 with a high passage number clustered with the hESC lines. The other hiPSC lines were equivalent to hESCs with regard to the expression of pluripotency markers, though other genes were overexpressed. hiPSC line gene expression profiles were also different from those of dermal fibroblasts, characterized by a lower expression level of all genes, except for *GATA2* and *RUNX1*.

To evaluate the functional significance of DNA methylation profiles, gene expression was determined for nine markers analysed for DNA methylation at TSS-associated CpG islands. Unmethylated DNA was predominant in the majority of genes in cell lines used for this analysis (S2 Fig) However, related genes were not expressed equally among samples, and *SOX17*, *T* and *FOXA2* were not detected in dermal fibroblasts. The intermediately methylated DNA detected in some hiPSC lines was not connected to any regular changes in the expression of related genes. No significant correlation was found between the level of DNA methylation and the relative expression of the adjacent gene.

## Discussion

Controversial questions regarding the equivalency of hiPSCs with hESCs are being widely discussed. In addition, genetic and epigenetic variations have been found among hPSC lines, and it is desirable to check and validate results for hPSCs on other hPSC lines in common use. Here, we analyze the DNA methylation profiles of TSS-associated CpG islands of pluripotency and differentiation markers, with a focus on the blood cell developmental lineage, to evaluate epigenetic variation in our hiPSC lines. hiPSC lines were derived from three different cell sources, including CD34^+^ cells obtained from PBMCs.

The DNA methylation profile was analysed using MethylScreen technology [28]. This technology combines the restriction from both methylation-sensitive and methylation-dependent restriction enzymes with qPCR to detect the sample DNA remaining after enzymatic treatment. The inclusion of two enzymes provides the density of methylcytosines in the DNA fragment. The detection of DNA methylation density is further supported by McrBC, which cuts the DNA at random sites within the vicinity of A/G^m^C [34]. Therefore, the assay can distinguish among cell populations with different loads of unmethylated, intermediately methylated and hypermethylated DNA [28]. Holemon *et al*. [28] reported that a 50:50 mixture of 100% methylated and entirely unmethylated samples can be represented as 50% of the hypermethylated DNA fraction and 50% of the unmethylated fraction and that the varying portion of intermediately methylated DNA fraction was due to the heterogeneously methylated population. In our study, samples with an increasing portion of methylated DNA were analysed for the markers *PAX6* and *TSPYL5*. Up to a 30% fraction of DNA was determined as being intermediately methylated, whereas the portion of unmethylated DNA was maintained. The sensitivity of the assay enabled monitoring of the DNA methylation density when more than 99% of the DNA population in the cell line assays was amenable to digestion.

Epigenetic reprogramming is a critical event during hiPSC derivation. The DNA methylation profiles of hiPSC lines shortly after derivation were compared with the control profile of hESCs to evaluate this phenomenon. hESCs were characterized by unmethylated DNA of all analysed genes (except *TSPYL5*), which corresponds to low DNA methylation levels in hESCs and the regulation of lineage control gene expression via post-translational modifications of histones and bivalent domains [18, 35–38]. An hESC-like DNA methylation profile was detected in hiPSC lines from NDFs, and hiPSC lines from adult cells were characterized by an increased fraction of methylated DNA. Such methylation was not detected in the source cells, suggesting that the modifications were induced during reprogramming. Consistent with our findings, global methylation in hiPSCs is greater than in source cells [20]. Reprogramming-induced DNA methylation is biased toward CpG islands [20] and contributes to the unique hiPSC DNA methylation profile along with retained somatic memory [22–24], which was not detected for the marker set analysed in this study. Using MethylScreen technology, hiPSC-specific aberrant DNA methylations were detected as intermediately methylated DNA, which does not reflect aberrant methylation of the entire DNA fragment in a portion of cells. But these methylation events consist of randomly methylated CpG dinucleotides of the analysed islands in a heterogeneous population of cells. In view of the higher degree of cellular heterogeneity in clonal hPSC cultures, especially hiPSCs derived by somatic cell reprogramming, the sensitivity of MethylScreen technology was shown to be amenable to the detection of intermediate methylation. Detected intermediately methylated DNA may be a natural part of the reprogramming process, while an excess of aberrant hypermethylation at early passages leads to the disappearance of hiPSCs in culture [39]. Individual hiPSC lines demonstrated variability in the level of intermediately methylated DNA, suggesting an association with the source cell type. NDF reprogramming by STEMCCA lentivirus generated hiPSCs with an hESC-like DNA methylation profile, while higher levels of intermediately methylated DNA was found in hiPSCs from adult source cells generated by both Sendai virus and episomal vector reprogramming. Nevertheless, differences among the level of aberrant DNA methylation in hiPSCs generated by different reprogramming methods was not confirmed due to sample variability. Among the cell lines used in this study the combination of STEMCCA lentivirus reprogramming of NDFs resulted in the most homogenous hiPSC population with the lowest load of aberrant DNA methylations in analysed markers. This may correspond to more effective hiPSC reprograming using integrating transgenes in ontogenetically younger NDF source cells. NDFs are most often used for iPSC derivation because of their higher reprogramming efficiency compared with other cell types. In contrast, hiPSC reprograming of adult source cells by non-integrating methods lead to a heterogeneous population with randomly methylated DNA that undergoes a longer selection process to achieve a homogeneous cell line.The effect of prolonged culturing on the hiPSC methylation profile was evaluated after thirty weeks of passage. After this period, hiPSCs normalized their DNA methylation profile toward an hESC-like one. Although the differences between hESCs and hiPSC decreased, persistent differences include intermediately methylated DNA for *UTF1* and *TSPYL5* (common to multiple hiPSC lines) and a hypermethylated DNA fraction for *TCERG1L* (some hiPSC lines). These findings are consistent with previous reports describing *TSPYL5* and *TCERG1L* as markers with different epigenetic statuses in hESCs and hiPSCs [13, 14, 20, 26, 40], whereas *UTF1* was newly identified in this study. Controversial observations have been reported for whole genome studies. Similar to our findings, continuous passage diminished the methylome differences between hESCs and hiPSCs [20]. On the contrary, the number of DNA aberrant methylations was not decreased, and the epigenetic resemblance to hESCs during passages was not reported for hiPSCs [12, 13]. The normalization of a DNA methylation profile in hiPSCs represents the reduction of *de novo* aberrant methylation and decreasing heterogeneity of the hiPSC population generated by the reprogramming process. During culturing, hiPSC colonies were manually passaged, and cells with hPSC morphologies were favored, shifting the cell population toward a homogeneous one. Acquired hESC-like DNA methylation profile in hiPSCs did not change during three weeks of hiPSC spontaneous differentiation. Therefore, the widespread intermediately methylated DNA of hiPSCs at early passages was not caused by spontaneous differentiation in culture.

The expression status of selected markers was determined for the hESC lines, hiPSC lines and dermal fibroblasts (both NDFs and ADFs). The resulting hierarchical clustering demonstrated that hiPSCs are distinct from both hESCs and dermal fibroblasts. The increased expression of all the genes (except *RUNX1* and *GATA2*) in hiPSCs compared with dermal fibroblasts indicated that these genes were activated during reprogramming. Pluripotency markers *POU5F1*, *SOX2* and *NANOG* were equally expressed between the hESC and hiPSC groups, though the expression of *UTF1* was higher in hiPSCs than in hESCs. *UTF1* is an early-stage indicator of successful somatic cell reprogramming, its activation is important for the derivation of mature hiPSCs, and it participates in the maintenance of bivalent gene expression [41, 42]. This present study identified *UTF1* as a new marker with a DNA methylation profile and expression pattern that can discriminate between hiPSCs and hESCs. The increased expression of all analysed differentiation markers distinguished hiPSCs from hESCs. Some markers decreased expression during prolonged hiPSCs culture, but they remained higher than in hESCs. Differential gene expression level of uniformly unmethylated DNA across the analysed cell lines and the lack of an effect of intermediately methylated DNA on gene expression were observed. Gene silencing is associated with region methylation density rather than a single CpG [43]. Therefore, because intermediate methylation represents a nonhomogeneous population with random methylation at single CpGs, this pattern may not effect gene expression. Moreover, hypomethylated regions are characterized by variable chromatin accessibility [44], suggesting that an unmethylated promotor region does not necessarily indicate active transcription of the adjacent gene. Taken together, despite the increasing resemblance of hiPSC and hESC DNA methylation profiles of selected pluripotency and differentiation markers during prolonged culture, hiPSCs are distinct from hESCs with respect to the expression of differentiation marker genes. These markers are highly activated during the reprogramming process, and their expression also remains higher compared to hESCs after the establishment of an hiPSC line. Gene expression was therefore not found to be associated with the DNA methylation profile, suggesting the involvement of other epigenetic modifications that affect chromatin accessibility.

## Conclusions

MethylScreen technology is a simple and reliable method for analysing the DNA methylation profile of CpG regions of genes of interest. Its detection capability enabled us to evaluate the degree of cellular heterogeneity within clonal hiPSC cultures. Using this technology, we determined that newly derived hiPSCs were characterized by a DNA methylation profile of selected pluripotency and differentiation markers that is dissimilar from both the respective source cells and hESCs. This included aberrant DNA methylation induced during somatic cell reprogramming, whereas retained somatic memory was not observed. hiPSC-specific aberrant DNA methylations were detected as intermediately methylated DNA, reflecting randomly methylated CpG sites in a heterogeneous hiPSC population. The level of aberrant methylation was associated with the type of source cells, and among the cell lines used in this study the lowest methylation load was found for hiPSCs generated from NDFs by STEMCCA lentivirus reprogramming. Regardless the level of aberrant methylation in low passage numbers all hiPSC lines normalized their DNA methylation profile toward a stable hESC-like one during prolonged culture, representing the decreasing heterogeneity of the hiPSC population. In contrast, expression status differences between hiPSCs and hESCs remained, indicating the involvement of gene expression-regulating mechanisms other than DNA methylation. *UTF1* was identified as a new marker with a DNA methylation profile and expression profile that can discriminate between hiPSCs and hESCs. MethylScreen technology together with the selected markers represents a new and affordable tool with the potential to evaluate and screen the process of the epigenetic establishment of newly derived hiPSC lines in individual laboratories. However, persisting gene expression differences between hiPSCs and hESCs must be considered before using these cells in downstream applications.

## Supporting Information

S1 Fig Characterization of CBIA hiPSC lines. (A) Immunofluorescence analysis of pluripotency markers performed on CBIA-1 hiPSCs at passage 6 with the indicated antibodies. Cells were fixed with 4% paraformaldehyde, permeabilized in 0.2% Triton X-100 and incubated with primary antibodies Oct-3/4 mouse IgG (Santa Cruz Biotechnology), Nanog rabbit IgG (Cell Signaling Technology, Danvers, MA), Sox2 mouse IgG and SSEA4 mouse IgG (both from R&D Systems, Minneapolis, MN) overnight. The antibodies were visualised by anti-rabbit and anti-mouse IgG Alexa Fluor 488 (Cell Signaling) and the nuclei were counterstained in 1 μg/mL Hoechst (BisBenzamide H33258, (Sigma-Aldrich, St. Louis, MO). The scale bar represents 100 μm. (B) In-vivo differentiation of CBIA-7 hiPSCs at passage 26 in teratomas. Differentiation of hiPSCs into all three germ layers was confirmed by the presence of glandular epithelia (endoderm), melanocytes (ectoderm), and mesenchymal cells (mesoderm). The teratoma assay was performed with the kind assistance of colleagues from the Department of Histology and Embryology, Faculty of Medicine, Masaryk University Brno. Cells were injected subcutaneously into immunodeficient mice and after 10 weeks, teratoma histology was analysed by hematoxylin/eosin staining.
(TIF)

S2 Fig Functional significance of the DNA methylation profile in the hPSC lines and dermal fibroblasts. Gene expression of selected markers (upper panels) is compared with a relevant DNA methylation profile (lower panels) for hESC lines, hiPSC lines, NDFs and ADFs. hiPSC lines in low (l.) and high (h.) passage numbers are presented. Gene expression values (mean + SD) are relative to the values from CCTL-14 set at 1; DNA methylation occupancy is reported as the percentage of unmethylated (blue box) and intermediately methylated (pink box) DNA.
(TIF)
