# Supplementary figures and images for: The Aberrant DNA Methylation Profile of Human Induced Pluripotent Stem Cells Is Connected to the Reprogramming Process and Is Normalized During In Vitro Culture
Source: PLoS One. 2016 Jun 23;11(6):e0157974. doi: 10.1371/journal.pone.0157974 (PMC4919089; doi:10.1371/journal.pone.0157974)

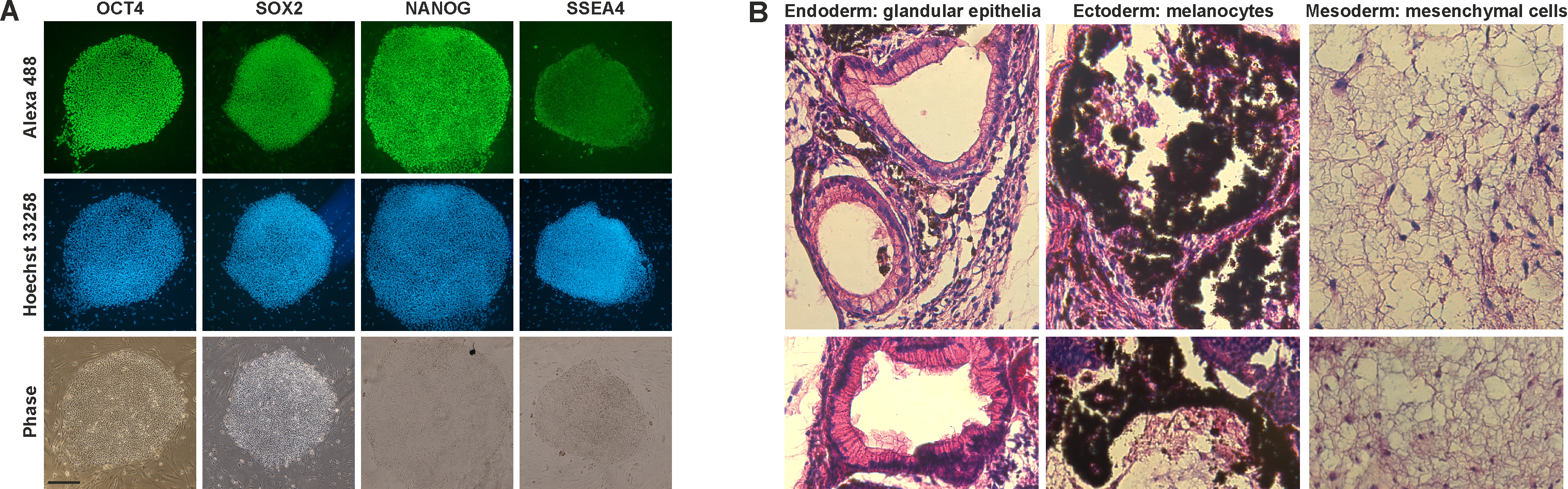

Supplement: S1 Fig — (A) Immunofluorescence analysis of pluripotency markers performed on CBIA-1 hiPSCs at passage 6 with the indicated antibodies. Cells were fixed with 4% paraformaldehyde, permeabilized in 0.2% Triton X-100 and incubated with primary antibodies Oct-3/4 mouse IgG (Santa Cruz Biotechnology), Nanog rabbit IgG (Cell Signaling Technology, Danvers, MA), Sox2 mouse IgG and SSEA4 mouse IgG (both from R&D Systems, Minneapolis, MN) overnight. The antibodies were visualised by anti-rabbit and anti-mouse IgG Alexa Fluor 488 (Cell Signaling) and the nuclei were counterstained in 1 μg/mL Hoechst (BisBenzamide H33258, (Sigma-Aldrich, St. Louis, MO). The scale bar represents 100 μm. (B) In-vivo differentiation of CBIA-7 hiPSCs at passage 26 in teratomas. Differentiation of hiPSCs into all three germ layers was confirmed by the presence of glandular epithelia (endoderm), melanocytes (ectoderm), and mesenchymal cells (mesoderm). The teratoma assay was performed with the kind assistance of colleagues from the Department of Histology and Embryology, Faculty of Medicine, Masaryk University Brno. Cells were injected subcutaneously into immunodeficient mice and after 10 weeks, teratoma histology was analysed by hematoxylin/eosin staining. (TIF) [file pone.0157974.s001.tif]

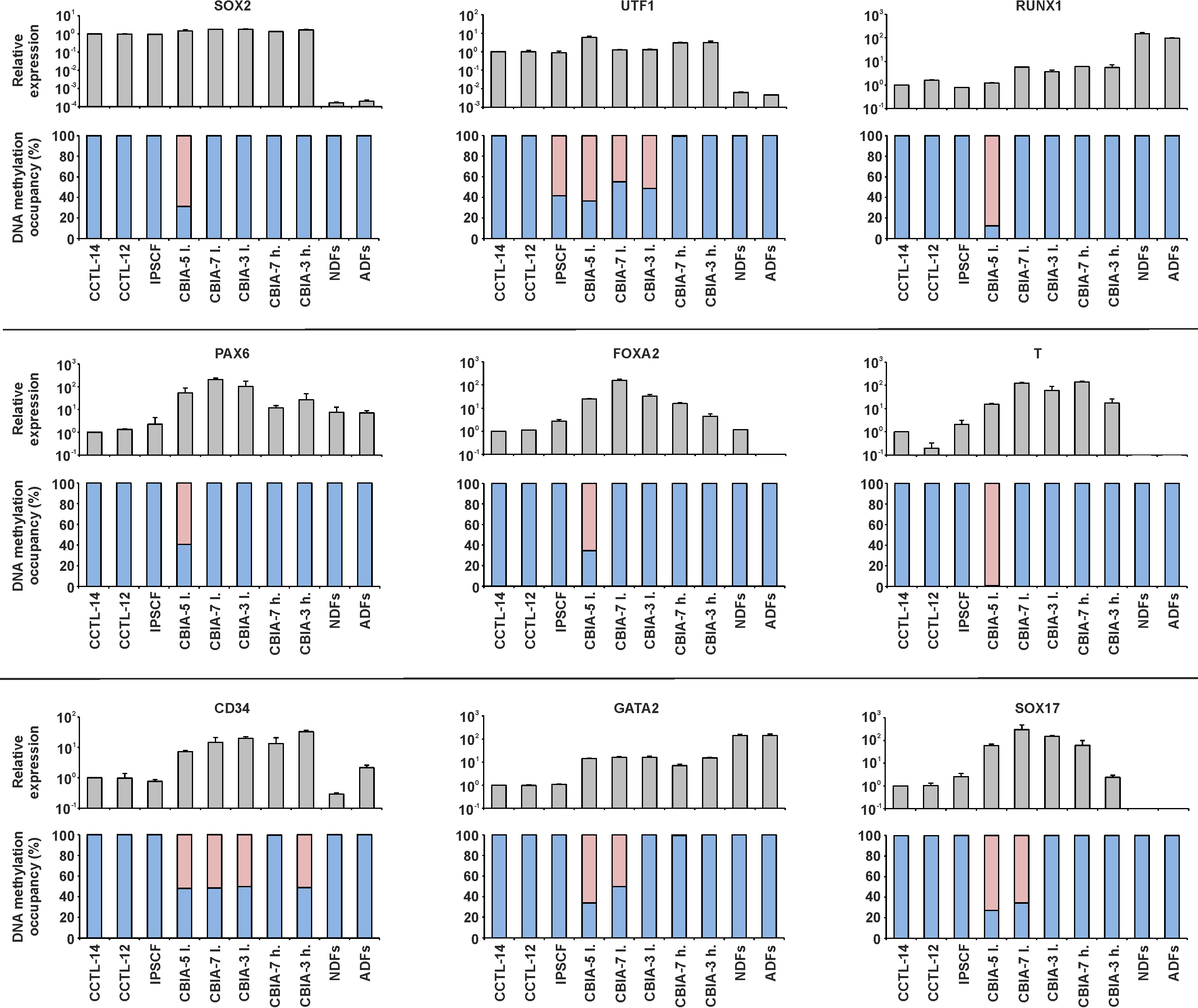

Supplement: S2 Fig — Gene expression of selected markers (upper panels) is compared with a relevant DNA methylation profile (lower panels) for hESC lines, hiPSC lines, NDFs and ADFs. hiPSC lines in low (l.) and high (h.) passage numbers are presented. Gene expression values (mean + SD) are relative to the values from CCTL-14 set at 1; DNA methylation occupancy is reported as the percentage of unmethylated (blue box) and intermediately methylated (pink box) DNA. (TIF) [file pone.0157974.s002.tif]
